# Association of the 12-Gene Breast DCIS Score^®^ Assay With Local Recurrence in Patients With Ductal Carcinoma *In Situ* Treated on Accelerated Partial Breast Radiotherapy Protocols

**DOI:** 10.3389/fonc.2021.671047

**Published:** 2021-06-17

**Authors:** Charles E. Leonard, Shannon P. Tole, Michelle P. Turner, John P. Bennett, Kathryn T. Howell, Dennis L. Carter

**Affiliations:** ^1^ Radiation Oncology, Rocky Mountain Cancer Centers, Littleton, CO, United States; ^2^ Biostatistics, Exact Sciences Corporation, Redwood City, CA, United States; ^3^ Radiation Oncology, Rocky Mountain Cancer Centers, Aurora, CO, United States

**Keywords:** breast cancer, radiotherapy, local recurrence, breast ductal carcinoma *in situ*, partial breast external beam radiotherapy

## Abstract

**Background:**

The following analysis explores clinicopathologic factors and the 12-gene Breast DCIS Score test result in order to better define an appropriate DCIS (ductal carcinoma *in situ*) population eligible for APBI (accelerated partial breast radiotherapy).

**Methods:**

This exploratory analysis aimed to retrospectively measure the association between the 12-gene Oncotype DX Breast DCIS Score^®^ assay (Redwood City, CA) and relevant clinicopathologic factors with locoregional recurrence in a pooled cohort of women treated with local excision and APBI on prospective phase II (NCT01185145) and phase III (NCT01185132) clinical trials. Univariable Cox proportional hazards regression was used to determine whether there was an association between local recurrence and DCIS Score result risk group (≥ 39 *vs* < 39) and clinicopathologic factors.

**Results:**

This analysis included 104 evaluable patients (n = 18 from NCT01185145 and n = 86 from NCT01185132). The median age was 60 years (range: 40-79). Seventy-nine percent of patients were postmenopausal. The median span of DCIS was 10 mm (range 2-45 mm). Two-thirds of the cohort presented with necrosis (71%). The distribution of DCIS Score^®^ results ranged from 0 to 82, with 69% of patients having a DCIS Score result < 39. The median follow-up time was 8.2 years in NCT01185145 versus 3.0 years in NCT01185132. There were 6 local ipsilateral breast recurrences. DCIS Score result was significantly associated with local recurrence in univariable modeling, hazard ratio = 10.3 (95% CI 1.7, 198.4); p = 0.010. None of the clinicopathologic characteristics resulted in any significant association with locoregional recurrence.

**Conclusion:**

The Breast DCIS Score assay demonstrated risk stratification in this cohort of patients treated with local excision and APBI pooled from two clinical trials. These results are consistent with those recently published utilizing whole breast radiotherapy. Due to the small number of local recurrence events and limited follow-up time, further investigations are needed to confirm findings.

## Introduction

Ductal carcinoma *in situ* (DCIS) is a proliferation of malignant epithelial cells of the ducts and terminal lobular units of the breast that do not invade the basement membrane. The incidence of DCIS has increased markedly since the early 1980s probably due to the adoption of screening mammography (1). Whole breast and interstitial radiotherapy has largely been shown to benefit patients and has historically been standard of practice to treat breast DCIS after lumpectomy (2–9). Several trials have demonstrated that breast radiotherapy might be omitted as an adjuvant treatment in some DCIS patients with acceptably low recurrence risks (10, 11).

The use of whole breast radiotherapy has been based on multiple trials showing a reduction in local recurrence but no improvement in survival. However, whole breast radiotherapy probably over-treats most patients. Newer strategies such as accelerated partial breast irradiation (APBI) have been utilized increasingly in patients considered to have a lower risk of local recurrence, especially those with breast DCIS. The updated American Society of Radiation Oncology (ASTRO) APBI guidelines have included “low risk” DCIS (defined by RTOG 9804 criteria) as suitable candidates for APBI (12, 13).

Estimation of the risk of local recurrence after lumpectomy alone has been based on traditional clinicopathologic factors, such as patient age at diagnosis, DCIS grade, and tumor size. These factors were derived from clinical trials, population studies and other tools of risk calculation (14, 15). The 12-gene Oncotype DX Breast DCIS Score^®^ assay has been clinically validated to provide individual 10-year local recurrence risk estimates after breast conserving surgery (BCS) alone (16, 17). The assay measures the RNA expression of 12 genes using RT-PCR in formalin-fixed paraffin-embedded tumor tissue. These studies also reported the significant correlation between the DCIS Score result and the risk of ipsilateral breast recurrence in DCIS patients after lumpectomy alone and the DCIS Score result maintained this significance even when other clinicopathologic factors are considered (16, 17). Data from the two validation studies (E5194 and Ontario DCIS Cohort) were combined in a patient-specific meta-analysis that adjusted for pre-specified clinicopathologic factors (age, tumor size, and year of diagnosis) to provide refined estimates of the 10-year risk of local recurrence after BCS alone (18).

The objective of the current analysis was to assess the correlation between the DCIS Score result and other clinicopathologic factors in the outcomes of women who underwent APBI on two prospective protocols. The hypothesis was that the DCIS Score result might be more informative than clinicopathologic factors alone in identifying patients with high risk disease who may not be “low-risk” candidates for APBI as described in ASTRO eligibility criteria.

## Methods and Materials

An exploratory analysis was retrospectively performed to measure the association between clinicopathologic factors and the DCIS Score result and the risk of any local *in situ* or invasive recurrence. All patients were enrolled with signed consent on one of two treatment protocols: a prospective phase II (NCT01185145; WIRB 20040075; principal investigator, Dennis Carter) and phase III (NCT01185132; WIRB #20091193; principal investigator, Charles Leonard) clinical trials conducted by Rocky Mountain Cancer Centers (RMCC/US Oncology) and treated with local excision followed by APBI. All experimental protocol methods were well documented and according to Western IRB protocol specifications, guidelines and regulations.

Estrogen receptor (ER) status, progesterone receptor (PR) status, and nuclear grade were determined centrally. Multifocal tumors were described only by local pathology and not determined or defined centrally. The presence of comedo necrosis was restricted to lesions with centrally necrotic ducts distended by cells exhibiting a pattern of growth consistent with ductal carcinoma *in situ*. The Breast DCIS Score assay was performed by quantitative RT-PCR on formalin-fixed paraffin-embedded DCIS tumor specimens in a central laboratory (Genomic Health, Inc., Redwood City, California) (16). Descriptive statistics and assay results were derived both overall and by clinical trial cohort. Kaplan-Meier estimates of the risk of local recurrence were generated and a log rank test was performed to compare survival distributions after stratifying by DCIS Score risk groups. Because of the small number of events, the intermediate and high risk groups were combined into a single category (DCIS Score result ≥ 39) and compared with the low risk group (DCIS Score result < 39). Univariable Cox proportional hazards models were used to determine whether there was an association between local recurrence and categorized DCIS Score risk group or other clinicopathologic factors on the pooled cohort of clinical trial patients. The number of local recurrence events was expected to be low; therefore, the pre-specified analysis plan called for univariable analyses only. In order to reduce bias in the Cox model parameter estimates, Firth’s correction was used (19). Due to the small sample size, profile-likelihood confidence intervals are reported (20). The data analysis was performed using SAS software, Version 9.4 of the SAS System for Windows. Graphics were created in SAS and R version 3.5.1 using the ggplot2 package (21, 22).

## Results

**Figure 1:**
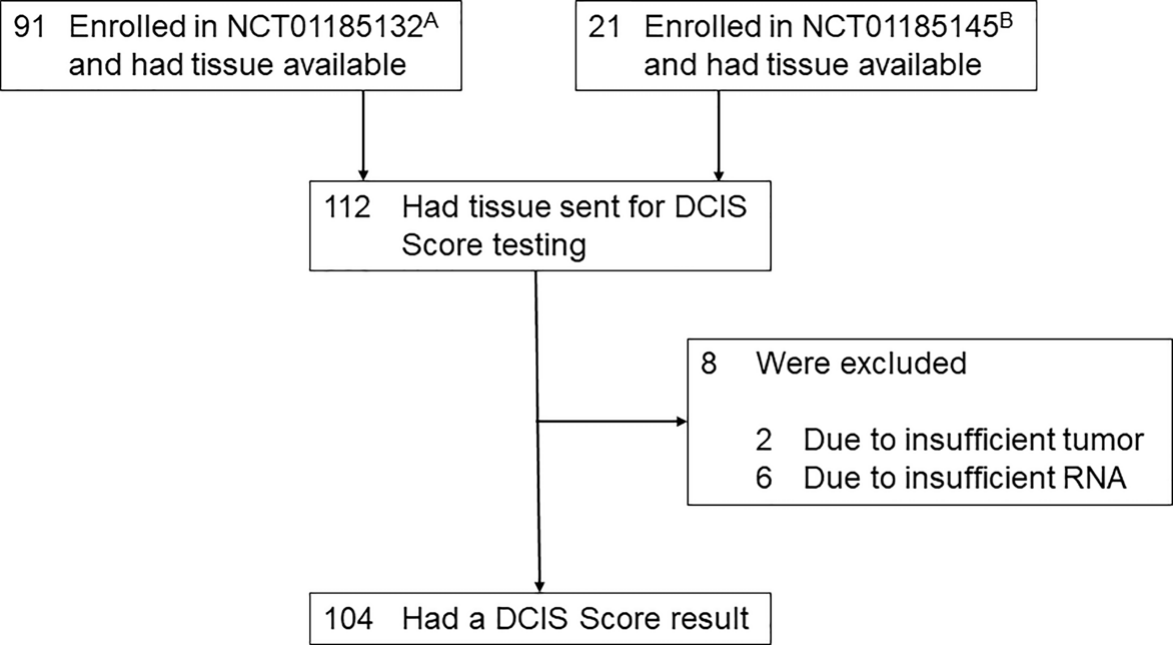
Study flow diagram (N=104). **(A)** A Phase III Randomized Study Comparing Intensity Modulated Planning *Versus* 3-Dimensional Planning for Accelerated Partial Breast Radiotherapy (2009-APBI); NCT01185132; WIRB #20091193. **(B)** A Phase II Accelerated Partial Breast Radiotherapy With Either Mammosite or Intensity Modulated Radiotherapy (APBI); NCT01185145; WIRB 20040075.

The DCIS tissue from 112 patients was available for the 12-gene assay (**Figure 1**). Twenty-one patients had been enrolled into the phase II trial (NCT01185145) and 91 had been enrolled into the phase III trial (NCT01185132). Eight patients were excluded, 2 due to insufficient tumor and 6 due to insufficient RNA. The final analysis included 104 evaluable patients enrolled in both studies (18 from the phase II study and 86 from the phase III study).

Baseline characteristics of the combined cohort are presented in **Table 1**. The median age was 60 years (range: 40-79). Seventy-nine percent of patients were postmenopausal. The median span of DCIS was 10 mm (range 2-45 mm). The median phase II study follow-up time of 8.2 years was longer than the 3.0 years in the phase III study. Over two-thirds of the cohort presented with necrosis (71%). The distribution of DCIS Score results ranged from 0 to 82. Sixty-nine percent of patients had a DCIS Score result < 39. There were differences between the two studies, however. The phase II study, which comprised approximately 17% of the patients in the entire cohort analyzed, notably included a younger cohort of patients and a slightly lower percentage of ER+ patients than the phase III study. The phase III trial included a higher proportion of patients with a DCIS Score result < 39 than the phase II trial.

**Table 1:** Patient characteristics, overall and by clinical trial.

	Overall (N = 104)	NCT01185132 (Phase III study) (n = 86)	NCT01185145 (Phase II study) (n = 18)
Age at diagnosis, years			
Median (range)	60 (40-79)	61 (40-79)	57 (44-66)
40-49	16 (15%)	10 (12%)	6 (33%)
50-59	34 (33%)	28 (33%)	6 (33%)
60-69	40 (38%)	34 (40%)	6 (33%)
70-79	13 (13%)	13 (15%)	0
Missing	1 (1%)	1 (1%)	0
Menopausal status			
Pre	22 (21%)	16 (19%)	6 (33%)
Post	82 (79%)	70 (81%)	12 (67%)
ER status			
Negative	10 (10%)	7 (8%)	3 (17%)
Positive	94 (90%)	79 (92%)	15 (83%)
PR status			
Negative	20 (19%)	12 (14%)	8 (44%)
Positive	84 (81%)	74 (86%)	10 (56%)
Nuclear grade			
1	7 (7%)	7 (8%)	0
2	56 (54%)	46 (53%)	10 (56%)
3	38 (37%)	31 (36%)	7 (39%)
Missing	3 (3%)	2 (2%)	1 (6%)
Comedo necrosis			
Not present	30 (29%)	26 (30%)	4 (22%)
Present	74 (71%)	60 (70%)	14 (78%)
Size (span of DCIS), mm			
Median (range)	10 (2-45)	10 (3-45)	9 (2-40)
≤10	54 (52%)	44 (51%)	10 (56%)
10 to 25	40 (38%)	35 (41%)	5 (28%)
>25	10 (10%)	7 (8%)	3 (17%)
Multifocality			
No	95 (91%)	83 (97%)	12 (67%)
Yes	9 (9%)	3 (3%)	6 (33%)
Margin width, mm			
Median (range)	7 (0-85)	5 (0-85)	8 (2-11)
≥10	36 (35%)	29 (34%)	7 (39%)
5 to <10	29 (28%)	23 (27%)	6 (33%)
3 to <5	26 (25%)	23 (27%)	3 (17%)
<3	13 (13%)	11 (13%)	2 (11%)
DCIS Score result			
Median (range)	27 (0-82)	25 (0-82)	38 (8-79)
<39	72 (69%)	63 (73%)	9 (50%)
≥39	32 (31%)	23 (27%)	9 (50%)
Follow-up time since DCIS diagnosis, years			
Median (range)	4.2 (0.6-10.2)	3.0 (0.6-7.6)	8.2 (1.5-10.2)

ER, estrogen receptor; PR, progesterone receptor.

**Figure 2:**
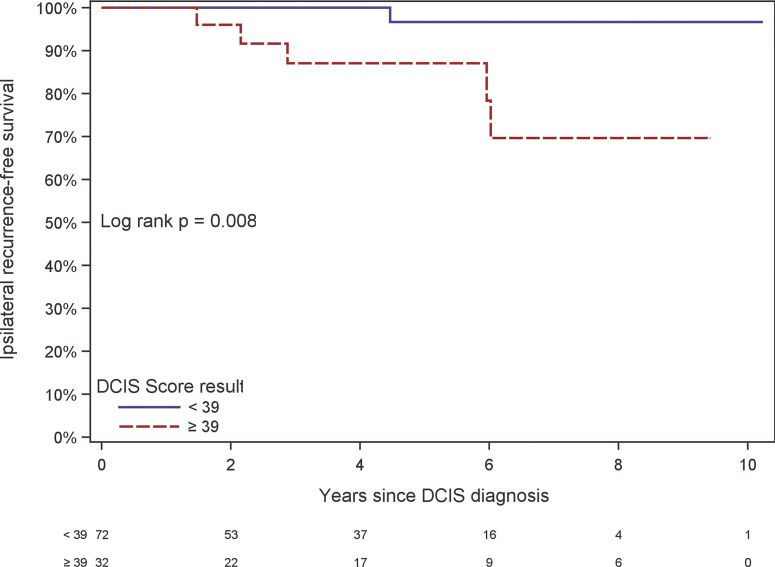
Kaplan-Meier plot of ipsilateral recurrence-free survival by DCIS Score result. The table at the bottom of the figure shows the number at risk in even-numbered years since DCIS diagnosis.

**Figure 3:**
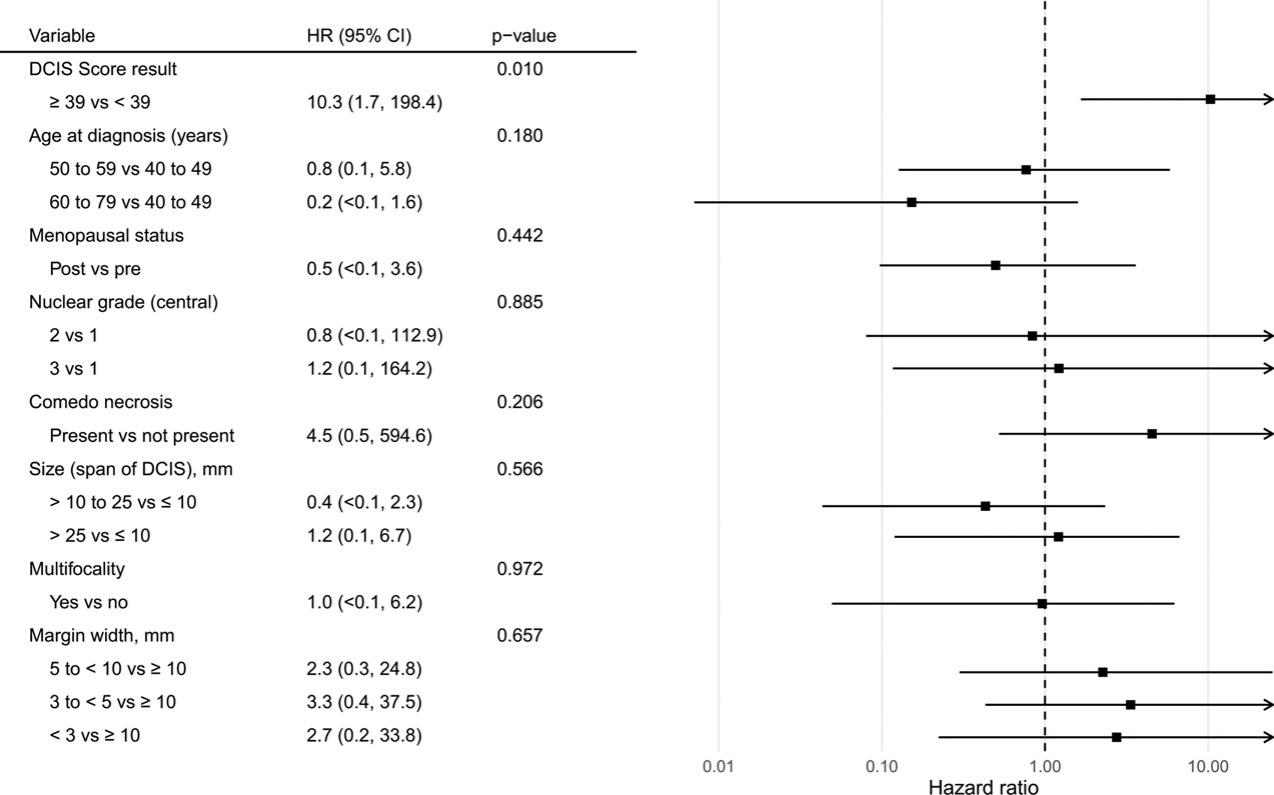
Forest plot of univariable Cox proportional hazards models for risk of ipsilateral recurrence by the DCIS Score result and relevant demographic, clinical, and pathologic features.

There were 6 local recurrences: 1 in the subgroup of patients with DCIS Score result < 39 (from the phase II trial) and 5 in the subgroup of patients with DCIS Score result ≥ 39 (3 from the phase III trial and 2 from the phase II trial). The Kaplan-Meier curves are displayed in **Figure 2**. There was a statistically significant difference in the risk of local recurrence stratified by DCIS Score result (p = 0.008). A forest plot illustrates the result of the univariable Cox proportional hazards models for risk of local recurrence (**Figure 3**). The DCIS Score result was significantly associated with local recurrence in univariable modeling [hazard ratio [HR] = 10.3 (95% CI 1.7, 198.4); p=0.010]. None of the clinicopathologic factors examined, such as age at diagnosis, menopausal status, central nuclear grading, presence of comedo necrosis, size, multifocality or margin width resulted in any significant correlation with locoregional recurrence. All results were highly variable due to the small number of events.

## Discussion

A cohort of 104 patients with DCIS treated with APBI from two clinical trials was analyzed retrospectively to determine the utility of the DCIS Score result in identifying patients who may not be appropriate for APBI. Although the DCIS Score result has been shown to correlate significantly with local recurrence probability after lumpectomy only, there has only been limited evidence to suggest that the same correlation is true for patients who have had post-lumpectomy whole breast radiotherapy. This may be especially pertinent since ASTRO guidelines have recognized the appropriate use of APBI in lower risk DCIS without definitively specifying how objectively and consistently to classify DCIS patients. There have been published reports exploring variables which result might be prognostic for local recurrence. Meatini et al. found that postmenopausal status, ER positivity and >1mm surgical margins were favorable factors for local control (23).

Previously published studies stratified DCIS Score results into low risk (DCIS Score result < 39), intermediate risk (DCIS Score result between 39 and 54), and high risk (DCIS Score result ≥ 55) (15). These three risk groups have estimated 10-year risks of local recurrence of 11%, 27% and 26%. After stratifying patients into the low risk and combined intermediate and high risk groups, we found that 5 of the 6 local recurrences were in the intermediate and high risk groups.

Rakovitch et al. reported seminal information concerning post-lumpectomy whole breast radiotherapy (24). In their cohort of 689 lumpectomy patients with adjuvant whole breast radiotherapy, there was significant stratification of patients into three groups by increasing local recurrence risk. Low, intermediate and high risk DCIS Score result risk groups (< 39, 39-54, > 54) resulted in 7.5%, 13.6% and 20.5% 10 year ipsilateral breast recurrence (p < 0.001).

A higher local recurrence risk has been presented in several other reports. Nineteen percent and 20% of patients with poorly differentiated or solid/comedo type ductal carcinoma *in situ* in the EORTC study respectively failed locally after breast conservation surgery and whole breast radiotherapy (6). The Early Breast Cancer Trialists’ Collaborative Group overview reported that roughly 17%-18% of patients with either high histologic or nuclear grade experienced local recurrence after conservative surgery and whole breast radiotherapy. Nineteen percent of patients with comedo/solid architecture present failed after similar treatment (25).

In contrast to the findings of the DCIS Score result, there was no apparent association between recurrence and any of the clinicopathologic factors (age, size, margin width, comedo necrosis or grade). While the number of recurrence events is small, there is a strong association between the DCIS Score result and recurrence risk that is not apparent when assessing risk with the traditional clinicopathologic factors. In the group with a DCIS Recurrence Score of ≥39 there were a total of 5 recurrences at a median of 2.9 years and 2 of these were in the group with scores ≥55 at a median of 1.8 years (26). There was only one recurrence with a score of <39 which occurred at 4.5 years. It is very probable that longer follow-up might result in more recurrences, especially in the <39 DCIS Recurrence Score group. However, our preliminary results do suggest that the DCIS Recurrence Score might be able to better stratify “low risk” patients who might be eligible for accelerated partial breast radiotherapy. As well, it could be useful for identifying patients with higher risk of recurrence who may not be appropriate “low risk” candidates for APBI as defined by ASTRO guidelines. Other investigators have also reported that improved methodologies of personalized strategies could contribute to our understanding of how accelerated partial breast radiotherapy could best be implemented for the care of DCIS patients (27).

Since the analysis cohort in this exploratory study was mostly from the phase III study, the small number of local recurrence events and limited follow-up time in the phase III trial, caution should be taken when interpreting the results. Hopefully, these results could interest investigators to pursue inquiries of larger patient numbers and longer follow-up to confirm these findings and improve overall outcomes for women with DCIS.

## Data Availability Statement

The raw data supporting the conclusions of this article will be made available by the authors, without undue reservation.

## Ethics Statement

The studies involving human participants were reviewed and approved by Western Institutional Review Board. The patients/participants provided their written informed consent to participate in this study.

## Author Contributions

ST contributed to data acquisition CL, MT, and JB contributed to data analysis. CL, MT, JB, KH, and DC contributed to manuscript writing and preparation. All authors contributed to the article and approved the submitted version.

## Conflict of Interest

MT and JB are employed by and have stock ownership in Exact Sciences Corporation. 

The remaining authors declare that the research was conducted in the absence of any commercial or financial relationships that could be construed as a potential conflict of interest.
